# The DNRA-Denitrification Dichotomy Differentiates Nitrogen Transformation Pathways in Mountain Lake Benthic Habitats

**DOI:** 10.3389/fmicb.2019.01229

**Published:** 2019-06-04

**Authors:** Carlos Palacin-Lizarbe, Lluís Camarero, Sara Hallin, Christopher M. Jones, Joan Cáliz, Emilio O. Casamayor, Jordi Catalan

**Affiliations:** ^1^Centro de Investigación Ecológica y Aplicaciones Forestales, Cerdanyola del Vallès, Spain; ^2^Center for Advanced Studies of Blanes, (CEAB–CSIC), Girona, Spain; ^3^Department of Forest Mycology and Plant Pathology, Swedish University of Agricultural Sciences, Uppsala, Sweden; ^4^Consejo Superior de Investigaciones Científicas, Cerdanyola del Vallès, Spain

**Keywords:** denitrification, DNRA, lithic biofilms, mountain lake, nitrogen deposition, remote ecosystems, sediment, 16S

## Abstract

Effects of nitrogen (N) deposition on microbially-driven processes in oligotrophic freshwater ecosystems are poorly understood. We quantified guilds in the main N-transformation pathways in benthic habitats of 11 mountain lakes along a dissolved inorganic nitrogen gradient. The genes involved in denitrification (*nirS*, *nirK*, *nosZ*), nitrification (archaeal and bacterial *amoA*), dissimilatory nitrate reduction to ammonium (DNRA, *nrfA*) and anaerobic ammonium oxidation (anammox, *hdh*) were quantified, and the bacterial 16S rRNA gene was sequenced. The dominant pathways and associated bacterial communities defined four main N-transforming clusters that differed across habitat types. DNRA dominated in the sediments, except in the upper layers of more productive lakes where *nirS* denitrifiers prevailed with potential N_2_O release. Loss as N_2_ was more likely in lithic biofilms, as indicated by the higher *hdh* and *nosZ* abundances. Archaeal ammonia oxidisers predominated in the isoetid rhizosphere and rocky littoral sediments, suggesting nitrifying hotspots. Overall, we observed a change in potential for reactive N recycling via DNRA to N losses via denitrification as lake productivity increases in oligotrophic mountain lakes. Thus, N deposition results in a shift in genetic potential from an internal N accumulation to an atmospheric release in the respective lake systems, with increased risk for N_2_O emissions from productive lakes.

## Introduction

According to the planetary boundaries framework (Rockström et al., 2009), anthropogenic alteration of the nitrogen (N) cycle is one of the major challenges facing the Earth system. Human activities have at least doubled the levels of reactive N (N_r_) available in the biosphere (Erisman et al., 2011), resulting in deposition of N_r_ in or near heavily populated areas as well as remote ecosystems (Catalan et al., 2013). In the context of global change, remote ecosystems — defined here as being affected by atmospheric processes rather than direct human action in catchment areas — can be particularly informative about potential large-scale changes in the Earth system (Catalan et al., 2013). Alpine lakes of the Northern hemisphere and subarctic regions are examples of remote ecosystems that have been exposed to increased N_r_ deposition during the last decades (Holtgrieve et al., 2011; Camarero, 2017), triggering a nutrient imbalance in these freshwater systems which are otherwise known to have low nutrient availability (Catalan et al., 2006). While alpine and subarctic lakes are often considered important sensors of global change (Smol, 2012), there is minimal understanding of how increased N_r_ availability affects microbially-driven N-cycle pathways in these ecosystems (McCrackin and Elser, 2010; Palacin-Lizarbe et al., 2018).

The N cycle is best described as a modular and complex network of biological N-transformation reactions carried out by metabolically versatile communities of microorganisms (Graf et al., 2014; Kuypers et al., 2018), whose overall composition largely determines whether N_r_ is lost, via denitrification or anammox, or retained in the system via dissimilatory nitrate reduction to ammonium (DNRA). Within lakes, benthic habitats are known as hotspots of N cycling due to steep redox gradients in the sediments and biofilms (Melton et al., 2014). Furthermore, the presence and composition of macrophytes also influence the biogeochemistry of the sediment (Gacia et al., 2009). In particular, isoetid species oxygenate the sediment and may promote coupled nitrification-denitrification (Vila-Costa et al., 2016). However, the effect of increased N deposition on the N-cycling microbial communities, and the factors controlling their distribution are poorly understood in mountain lakes.

**FIGURE 1:**
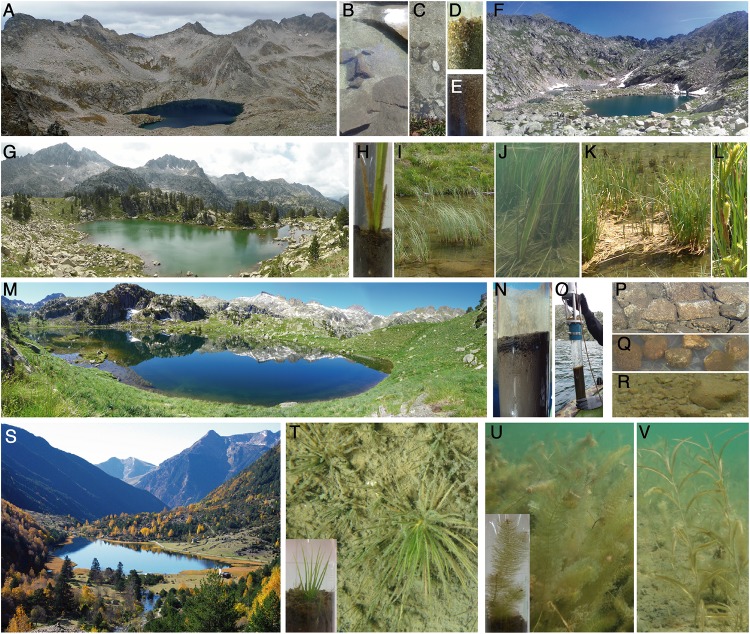
Examples of the lakes and habitats studied. Lakes: Contraix **(A)**, Gelats de Bergús **(F)**, Bassa de les Granotes **(G)**, Plan **(M)**, and Llebreta **(S)**. Benthic habitats: sediments near the deepest point of the lake (non-vegetated) **(N,O)**; littoral sediments from beds of isoetids [*Isoetes lacustris* **(T)**] and elodeids [*Myriophyllum alterniflorum* **(U)***, Potamogeton alpinus* **(V)**] macrophytes, helophyte (*Carex rostrata*) belts **(H–L)** and rocky areas **(B–E)**; and lithic biofilms from littoral cobbles **(P–R)**.

Our study aims to investigate how the distribution of microbial communities in general and those that drive different N-transformation pathways changes across a range of different benthic habitats in mountain lakes that have been affected by enhanced N deposition in the absence of significant acidification (Camarero and Catalan, 1998). We hypothesise that benthic habitat type and lake productivity together determines the fate of deposited N and that increased productivity will promote pathways resulting in N_r_ loss. Lakes at lower altitudes tend to be more productive, particularly if they are small since the productive period is longer (Catalan et al., 2009) and phosphorus loading to the lake increases as the catchment is more vegetated (Kopàček et al., 2011). In the Pyrenees, more than 70% of the lakes are considered ultraoligotrophic based on total phosphorus (TP; <150 nM), whereas 22 and 6% are oligotrophic and mesotrophic, respectively (Catalan et al., 2006). In general, more productive oligotrophic mountain lakes exhibit low dissolved inorganic nitrogen (DIN) concentrations due to higher consumption of excess N from atmospheric loading by primary producers (Camarero and Catalan, 2012). We therefore selected lakes to establish a DIN gradient and sampled lithic biofilms, sediments with elodeid, isoetid and helophyte macrophytes, and littoral and deep non-vegetated sediments (Figure 1). We then characterised the N-functional pathways by quantifying the abundances of key N-functional genes involved in denitrification, nitrification, DNRA and anammox pathways (Table 1). We also determined the bacterial community composition in the benthic habitats and linked these to the functional guilds using a multivariate approach combined with indicator species analyses. The environment was characterised by including proximal (benthic) and more distal (lake) descriptors to capture potential drivers acting at different spatial scales (Wallenstein et al., 2006; Battin et al., 2016).

**Table 1:** N-functional genes accounted for in this study.

Gene	Enzyme	Pathway	Reaction	Process type
*amoA*	Ammonium monooxygenase	Nitrification	Ammonium oxidation to hydroxylamine	Aerobic
*nirS*	Nitrite reductase (cytochrome-cd1)	Denitrification	Nitrite reduction to nitric oxide	Anaerobic
*nirK*	Nitrite reductase (copper-based)		
*nosZ*	Nitrous oxide reductase	Denitrification	Nitrous oxide reduction to dinitrogen	Anaerobic
*nrfA*	Nitrite reductase (formate-dependent)	DNRA	Nitrite reduction to ammonium	Anaerobic
*hdh*	Hydrazine dehydrogenase	Anammox	Hydrazine oxidation to dinitrogen	Anaerobic


## Materials and Methods

### Sampling Location and Habitat Description

The lakes are located in the central region of the Pyrenees mountain range within the Aigüestortes i Estany de Sant Maurici National Park (Table 2 and Figure 1). All lakes are dimictic and ultra-oligotrophic (TP < 150 nM) except for Bassa de les Granotes, which is classified as oligotrophic (150 < TP < 300 nM; Catalan et al., 1993) with a circumneutral pH (∼7; Vila-Costa et al., 2014). All main benthic habitats in the lakes were considered (Figure 1), although certain habitats were present in only a few lakes (Table 2 and Supplementary Table S1). Plan Lake is particularly rich in macrophytes, including isoetids (*Isoetes setacea, I. palustris*, and *Subularia aquatica*), elodeids (*Myriophyllum alterniflorum, Potamogeton alpinus*, and *P. berchtoldii*) and the helophyte *Carex rostrata* (Gacia et al., 1994). Sampling was carried out during the ice-free period (June–November) of 2013 and 2014, with a total of 30 sites and 226 samples analysed.

**Table 2:** Site location, habitats studied and characteristics sorted by the dissolved inorganic nitrogen (DIN) concentration.

Lake (Abbreviation)	Vegetation belt	Habitats Studied^a^	Latitude (N)	Longitude (E)	Altitude (m a.s.l.)	Area (ha)	Catchment (ha)	Depth^b^ (m)	Renewal time (months)	TP^c^ (nM)	DIN^d^ (μM)
Redon de Vilamòs (R)	Alpine	I	42.78078	0.76233	2209	0.6	12	5	1.7	NA	1.2
Plan (P)	Subalpine	D, I, E, C, L	42.62248	0.9307	2188	5	23	9	15.1	102	1.7 ± 0.9
Bassa de les Granotes (G)	Alpine	D, L	42.5733	0.97124	2330	0.7	3	5	9.9	292	2.4 ± 0.7
Redó Aigüestortes (RA)	Subalpine	D, L	42.58216	0.95949	2117	6.3	325	11	1.6	76	8.5 ± 0.9
Gelat de Bergús (GB)	Alpine	D, R, L	42.59106	0.96331	2493	1.4	24	8	2.3	42	8.8 ± 3.3
Llong (Lo)	Montane	D, R, L	42.57431	0.95063	2000	7.1	1111	13	0.6	89	10.3 ± 11.6
Bergús (B)	Alpine	D, L	42.58947	0.95717	2449	6.2	126	50	3.9	44	17.1 ± 11.3
Llebreta (Le)	Montane	D, C, R, L	42.55083	0.89031	1620	8	5438	12	0.1	89	17.9 ± 2.7
Contraix (C)	Alpine	D, R, L	42.58874	0.91861	2572	9.3	100	59	9.9	49	18.0 ± 1.3
Redon (RC)	Alpine	D, R, L	42.64208	0.77951	2235	24.1	153	73	36	58	23.5 ± 19.6
Pòdo (Po)	Alpine	D, L	42.60307	0.93906	2450	4.6	33	25	9.4	75	25.2 ± 14.7


*^a^D, sediments in the deepest point of the lake; I, isoetid littoral sediments; E, elodeid littoral sediments; C, *Carex rostrata* belts littoral sediments; R, rocky areas littoral sediments, L, lithic biofilms from littoral cobbles. ^*b*^Maximum water column depth. ^*c*^Total phosphorus (Camarero and Catalan, 2012). ^*d*^DIN concentration (sum of nitrate, nitrite and ammonium) in the overlying water.*

### Water, Lithic Biofilm, and Sediment Characterisation

The overlying water, sediments and lithic biofilms were characterised using physical, chemical and biological variables (Supplementary Table S1). The temperature of the overlying water was measured at the time of sampling. For chemical analyses, water samples were filtered through a pre-combusted (4 h at 450°C) GF/F glass fibre philtre. Nitrate and sulphate were determined by capillary electrophoresis using a Quanta 4000 (Waters) instrument. Ammonium and nitrite were determined by colourimetric methods in a segmented-flow autoanalyser (AA3HR, Seal), using the Berthelot reaction for ammonium (Bran+Luebbe method G-171-96) and the Griess reaction for nitrite (Bran+Luebbe method G-173-96). Dissolved organic carbon (DOC) was measured by catalytic combustion to CO_2_ and detection by IR spectroscopy in a TOC5000 (Shimadzu) analyser.

Lithic biofilms were sampled collecting several cobbles (ø ∼10 cm) from different sites of the lake. Cobbles were scraped entirely (upper and lower sides) with clean metal brushes and washed with deionized water and pooling together the collected material. Biofilm subsamples were collected on 0.2-μm pore polycarbonate membranes for DNA analysis, and triplicate volumes were filtered through a pre-combusted and pre-weighted GF/F glass fibre philtre for chemical and physical analyses. Sediment cores (ø 6.35 cm) were collected with a gravity corer (Glew, 1991) around the deepest point of each lake or manually by scuba diving for the littoral sediments. The cores were sliced in three sections (0–0.5, 0.5–2, and 2–4 cm) to capture the oxic and the nitrate reduction zones (Melton et al., 2014).

For total carbon (C) and N and isotopic composition, ca. 5 mg of the freeze-dried sample was placed with a catalyst (Va_2_O_5_) in tin capsules, and the analyses were performed by the University of California Davis Stable Isotope Facility. Organic matter (OM) content was determined using the loss on ignition (LOI) procedure (Heiri et al., 2001). The median grain size of the sediment was determined by laser diffraction (Mastersizer, 2000, Malvern Instruments Ltd., United Kingdom), using freeze-dried sediment rehydrated in distilled water and introduced into the sample dispersion unit (Hydro 2000 G, Malvern Instruments Ltd., United Kingdom) after adding hexametaphosphate and sonicating to avoid aggregates. Laser obscuration was between 10–20% and the measuring range between 0.02 and 2000 μm.

### DNA Extraction and Quantitative PCR of 16S rRNA and N Cycle Genes

DNA was extracted from 0.33 ± 0.06 g of sediment or lithic biofilm using the FastDNA^®^ Spin Kit for Soil (MP Biomedical) following the manufacturer’s instructions. The extracted DNA was quantified using the Qubit^®^ fluorometer (Thermo Fisher Scientific Inc.).

Quantitative real-time PCR (qPCR) was used to quantify functional genes encoding enzyme involved in N-cycle pathways (Table 1 and Supplementary Table S2), as well as the bacterial 16S rRNA gene. All qPCR reactions were performed in duplicate in a total reaction volume of 20 μL using DyNAmo Flash SYBR Green qPCR kit (Thermo Fisher Scientific Inc.), 0.1% Bovine Serum Albumin, 0.5–1.0 μM of each primer and 15 ng DNA on the Biorad CFX Connect Real-Time System. Primers (Rotthauwe et al., 1997; Hallin and Lindgren, 1999; Lopez-Gutierrez et al., 2004; Mohan et al., 2004; Throbäck et al., 2004; Henry et al., 2006; Schmid et al., 2008; Tourna et al., 2008; Jones et al., 2013; Welsh et al., 2014), amplification protocols and resulting efficiencies for each assay are listed in Supplementary Table S3. Potential inhibition of the PCR reactions was checked by amplifying a known amount of the pGEM-T plasmid (Promega) with the plasmid-specific T7 and SP6 primers added to the DNA extracts and non-template controls. No inhibition of the amplification reactions was detected with the amount of template DNA used. Standard curves for each assay were generated by serial dilutions of linearized plasmids with cloned fragments of the respective gene. Standard curves were linear (*R*^2^ = 0.997 ± 0.003) in the range used, and amplification efficiency was 90% for the 16S rRNA gene and 65–88% for the functional genes (Supplementary Table S3). Melting curve profiles were inspected, and final products were run on an agarose gel to confirm amplicon size. Non-template controls resulted in negligible values.

### Sequencing of the 16S rRNA Gene, Sequence Processing and OTU Clustering

The diversity and structure of total bacterial and archaeal communities were determined by targeting the V3-V4 region of the 16S rRNA gene (Takahashi et al., 2014). Amplicon libraries for each sample were generated using a two-step protocol (Berry et al., 2011). First, PCR products were generated in duplicate 20 μL reactions per sample using 16S rRNA primer constructs that included Nextera adapter sequences, with reactions consisting of Phusion PCR mastermix (Thermo Fisher Scientific), 0.5 μg μL^-1^ BSA, 0.25 μM of each primer and 10 ng extracted DNA. Thermal cycling was performed for 25 cycles, and cycling conditions and primer sequences are listed in Supplementary Table S3. The resulting PCR products were pooled and purified using the AMPure bead purification kit, and 3 μL of the purified product was used as template in the second PCR using barcodes. Duplicate 30 μL reactions were performed for each sample, with similar reagent concentrations as in the first step except for the use of 0.2 μM final primer concentrations. PCR was performed according to Supplementary Table S3. Products were pooled, bead purified, followed by equimolar pooling and sequencing performed by Microsynth AG (Balgach, Sweden) using the Illumina MiSeq platform with v3 chemistry (2 × 300 bp paired-end reads).

Paired-end reads were merged using PEAR (Zhang et al., 2013) and dereplicated and clustered into operational taxonomic units (OTUs) at a cut-off of 3% identity using UPARSE (Edgar, 2013). The final dataset comprised 13069 OTUs after removal of chimaeras and singletons, with 83% of the quality filtered sequence pool mapped back to OTUs. Taxonomic assignment was carried out with the RDP classifier (Wang et al., 2007) against the SILVA reference database (release 119) (Quast et al., 2013). Sequences classified as mitochondria or chloroplasts were excluded. The original OTU table was rarefied (100 random subsampling) to 10660 sequences per sample. The sequences are available in the NCBI Sequence Read Archive (PRJNA494630).

### Statistical Methods

All multivariate, clustering and correlation analyses were performed using R (R Core Team, 2017). Comparisons of gene abundances between habitat types were performed using Kruskal–Wallis (KW) and Wilcoxon–Mann–Whitney (WMW) tests. Principal component analysis (PCA) and Redundancy analysis (RDA) using Hellinger distances (Borcard et al., 2011) were used to investigate the unconstrained ordination of the relative abundances of the N-functional genes studied (PCA) and of the bacterial community composition (PCA), and the relationship between the relative abundance of the N-functional genes and the environmental conditions (RDA), as well as between the overall bacterial community composition and the relative abundance of the N-functional genes (RDA). Hereafter, we refer to the PCAs as gene-PCA (gen-PCA) and community-PCA (com-PCA), and to the RDAs as gene-environment-RDA (gen-env-RDA) and community-gene-RDA (com-gen-RDA), respectively. In the analyses, functional gene abundances were standardised to total 16S rRNA gene copy numbers. Taxa < 5% occurrence (3453 of the total 13069 OTUs) were excluded from the bacterial community composition analysis (com-PCA and com-gen-RDA), and values for nitrate, nitrite, ammonium and sulphate were log-transformed in the gen-env-RDA. In the RDAs, forward selection was used to identify a minimum set of significant explanatory variables (*p* < 0.05; Blanchet et al., 2008), which exhibited low collinearity (variance inflation factors well below 10). Permutation tests of the resulting ordinations showed significant pseudo*-F* values (*p* < 0.05, *n* = 1000) for the main explanatory axes in each ordination — first to third axes in the gen-env-RDA, and first to fifth axes in the com-gen-RDA.

A structure of four sample clusters was present in both RDAs. Consequently, we used the samples scores of the three main axes of the com-gen-RDA as coordinates in four-group *k*-means clustering. We looked for indicative OTUs of each cluster performing a multi-level pattern analysis using the *multipatt* function from the *indicspecies* R package (Cáceres and Legendre, 2009), considering site group combinations, and the entire OTU set (13069) as the community data table. For each OTU, the method provides an indicator value (IndVal) of each cluster or a joint set of them. We accepted as significant indicator taxa those with adjusted *p*-value < 0.001, using the false discovery rate method to calculate the adjusted *p*-value (Storey et al., 2004).

## Results

### Genetic Potentials

**FIGURE 2:**
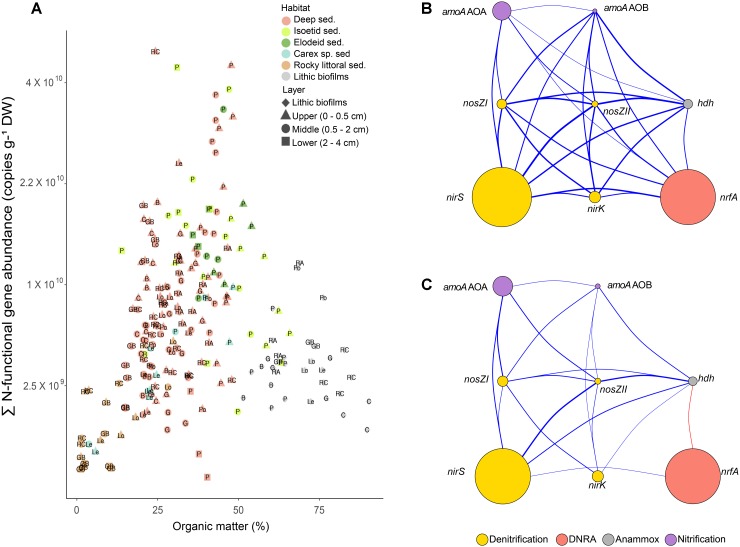
**(A)** Sum of the accounted N-functional gene copies g^-1^ of dry weight (DW) against the percentage of organic matter (OM). Labels are the Lake abbreviations (see Table 2). Note that *Y*-axis has a square root scale. **(B,C)** Correlation structure of N-transforming gene abundances in mountain lake benthic habitats. N-functional gene abundances are in gene copies g^-1^ DW **(B)** or standardised to the 16S rRNA gene copies **(C)** (Supplementary Table S2). The nodes symbolise the genes whose colour and size indicate their associated pathway and abundance, respectively. Link width is proportional to the coefficient values of significant associations (*p* < 0.05), and blue and red colours indicate positive and negative Spearman’s correlations, respectively.

The sum of the N-functional gene copies per dry weight of sediment increased with OM (*r* = 0.42, *p* < 0.001, Figure 2A) but with a large scattering. Individual gene abundances were highly positively correlated among them (Figure 2B). However, the correlation structure markedly simplified when standardising by the 16S rRNA copy number in each sample (Figure 2C), showing that the *nrfA* pool was weekly related to the rest of N-functional gene pools. The sum of the N-functional gene copies accounted for an average of 15 ± 8% (mean ± SD) of bacterial 16S rRNA gene copies across all samples (Supplementary Figure S1). Maximum values of 52% were found in the lower sediment layer (2–4 cm) near the isoetid rhizosphere, while minimum values of 2% were observed in lithic biofilms. Hereafter, unless otherwise indicated, we report the N-functional gene abundance standardised to total bacterial 16S rRNA gene copies.

**FIGURE 3:**
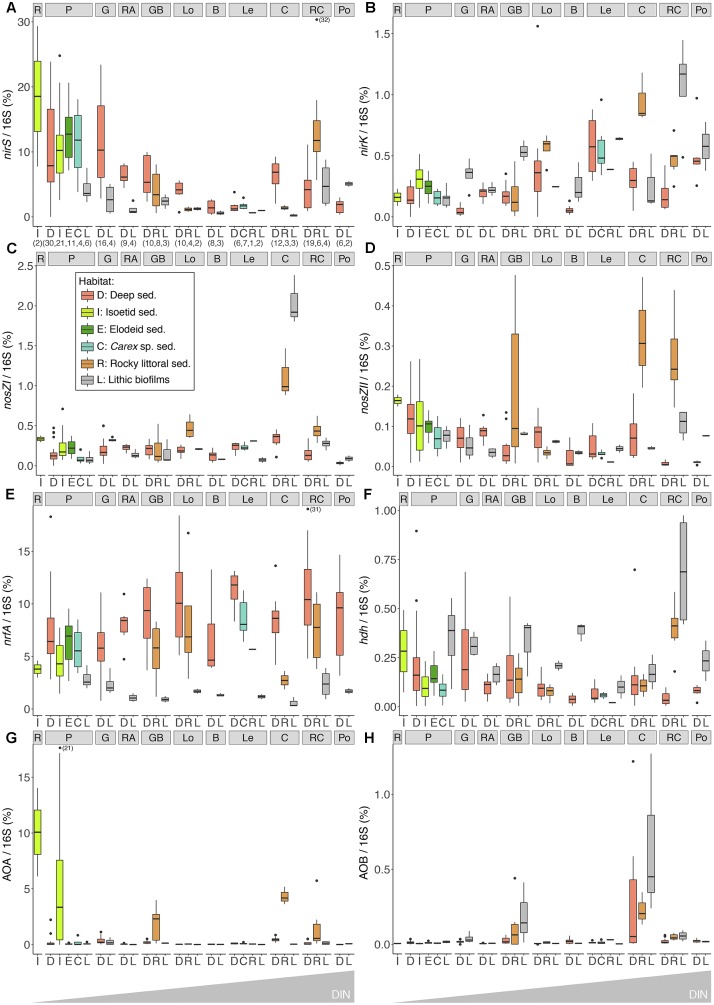
Abundances of *nirS* **(A)**, *nirK* **(B)**, *nosZ*I **(C)**, *nosZII* **(D)**, *nrfA* **(E)**, *hdh* **(F)**, archaeal *amoA* **(G)**, and bacterial *amoA* **(H)** standardised to the 16S rRNA gene copies in different lakes and habitats. Numbers in brackets in the *X*-axis indicated the samples analysed. Boxplots depict the interquartile range (box), median value (line), 1.5x interquartile (whiskers), and outliers (points). Lakes are arranged from left to right by increasing water-column DIN level (see Table 2 for lake abbreviations and characteristics).

The *nirS* and *nrfA* genes showed the highest relative abundances, up to 33 and 18% of total 16S gene copies, respectively. The abundance of *nirS* was approximately 50-fold greater than *nirK* across all lakes, with the highest numbers detected in the more productive lakes (R, P and G, Table 2 and Figure 3A,B). The abundance of *nirK* genes exhibited an overall trend of increasing abundance with lake DIN levels (Figure 3B), opposed to that observed for *nirS*. Higher *nrfA* abundance was observed in the sediments of the deepest part of the lakes (Figure 3E), while abundances in the elodeid sediments were significantly higher than those of the isoetids (KW test, *p* = 0.028). Closer inspection of the macrophyte sediment profiles showed a significant *nrfA* increase deeper in the elodeid sediments (*r* = 0.85, *p* < 0.001; Supplementary Figure S2A), while no trend was observed in the isoetid sediments.

The *amoA* gene of ammonia-oxidising archaea (AOA) was more abundant than the bacterial (AOB) counterpart. No obvious trend was observed for either AOA or AOB abundance across the lake DIN gradient. Although the average total abundance of ammonia oxidisers across all lakes was low relative to those of 16S rRNA genes (0.87 ± 2.66% and 0.05 ± 0.14% for AOA and AOB, respectively), several lakes showed AOA and AOB proportions of 57 and 23% of the total N-functional gene abundance. The highest AOA abundance was observed in the lower sediment layers of the isoetid rhizosphere and rocky littoral sediments of high-altitude lakes in the alpine belt, that is, those located above treeline (Figure 3G). Abundances of AOA were significantly higher in isoetid than elodeid sediments (KW test, *p* < 0.001), and increased with depth in the former (*r* = 0.64, *p* = 0.001; Supplementary Figure S2B). All habitats of the highest altitude lakes (GB and C lakes, Table 2 and Figure 3H) showed a relatively high abundance of AOB copies compared to the same habitats in lakes at lower elevations.

The gene variants of the nitrous oxide reductase, *nosZ* clade I and II, as well as the *hdh* gene associated with the anammox pathway, exhibited low relative abundances (Figure 3C,D,F). The abundance of clade I *nosZ* genes was typically higher (∼7-fold on average) than that of clade II *nosZ* across all lakes and habitats. The lithic biofilm habitats of Contraix, the most elevated lake, showed the highest abundance of *nosZ* clade I genes (Figure 3C), while *nosZ* clade II abundances were higher in the rocky littoral sediments of alpine lakes (Figure 3D). Relative abundances of *hdh* were higher in the lithic biofilms (Figure 3F), with no obvious relationship with DIN levels across the lakes.

### N-Functional Genes and the Environment

**FIGURE 4:**
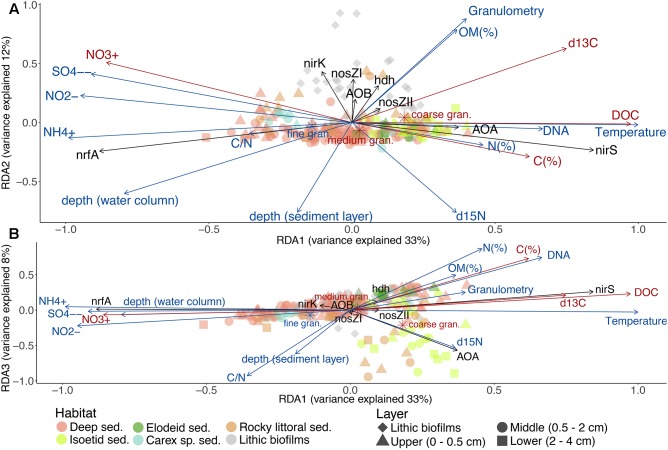
RDA based on N-functional gene abundances (response variables, black arrows) and the environmental variables (explanatory variables, blue arrows). Red arrows represent the unselected environmental variables in the previous forward selection. Triplots of the 1st and 2nd **(A)** or of the 1st and 3rd **(B)** main gradients. See Supplementary Figures S4A,B for samples scores. The axes sizes are proportional to the explained variation.

The constrained ordination of N-*functional* gene abundances identified three distinct gradients explaining 54% of the total variation across habitats and lakes (Figure 4). A similar result was obtained in a non-constrained analysis (Supplementary Figure S3), indicating that the main environmental drivers were captured by the constrained analysis. The main variation of benthic N-cycling genetic potentials was across a *nirS* vs *nrfA* abundance gradient (Figure 4). The *nirS*-rich samples corresponded to those from shallow and productive lakes (R, P, and G, Table 2 and Supplementary Figures S4A,B), specifically the upper sediments in all habitats and sediments near the roots of isoetids. These sites were associated with higher temperature, DNA content, isotopic signatures, DOC content, and C and N content, as well as coarser granulometry (Supplementary Figure S5) and lower C/N and nitrate/nitrite ratios (Figure 4). In contrast, *nrfA* rich environments occurred in the deep parts of the deep lakes, the lower layers of all sediments (except the isoetid rhizosphere) and the littoral sediments of the montane belt lakes (Table 2 and Supplementary Figures S4A,B). Sulphate, ammonium, nitrate and nitrite concentrations were higher in these sites compared to those associated with *nirS*. The same *nirS*-*nrfA* main axis was also found if only samples from the deep habitat were included in the analysis.

The gen-env-RDA second axis of variation discriminated between sediments and lithic biofilms. The latter characterised by higher abundances of *nirK*, *nosZI, nosZII*, *hdh* and AOB (Figure 4A). The rocky littoral sediments of Lake Contraix separated from the other sediment samples (Supplementary Figure S4A). These sites shared relatively high concentrations of nitrate, nitrite and sulphate in the overlying water, high OM content, and particular isotopic signatures (high δ^13^C and low δ^15^N). Finally, the third axis of variation was associated with the AOA abundance (Figure 4B) and resulted in the segregation of the majority of the isoetid sediments from the other habitats. The sites with the highest abundance of AOA were located close to the isoetid rhizosphere and in the rocky littoral sediments of the alpine lakes (Supplementary Figure S4B and Table 2). These samples showed high δ^15^N values and likely corresponded to more oxygenated sediments (Figure 4B).

### N-Functional Genes and the Associated Microbial Community

**FIGURE 5:**
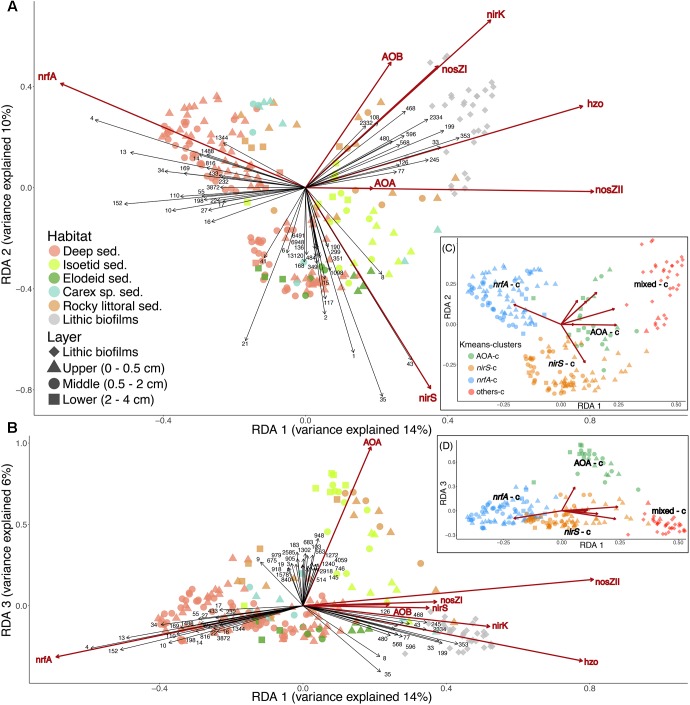
RDA with the OTUs abundance (response variables) and the abundance of the N-functional genes (explanatory variables, red arrows). Triplots of the 1st and 2nd **(A)** or the 1st and 3rd **(B)** main gradients. Only the most influential OTUs for each gradient are shown (black arrows and ID, Supplementary Table S6). Clusters are resulting from the k-means analysis with the sample scores of the three main RDA gradients (Subplots C and D). See Supplementary Figures S4C,D for samples scores. The axes sizes are proportional to the explained variation.

**FIGURE 6:**
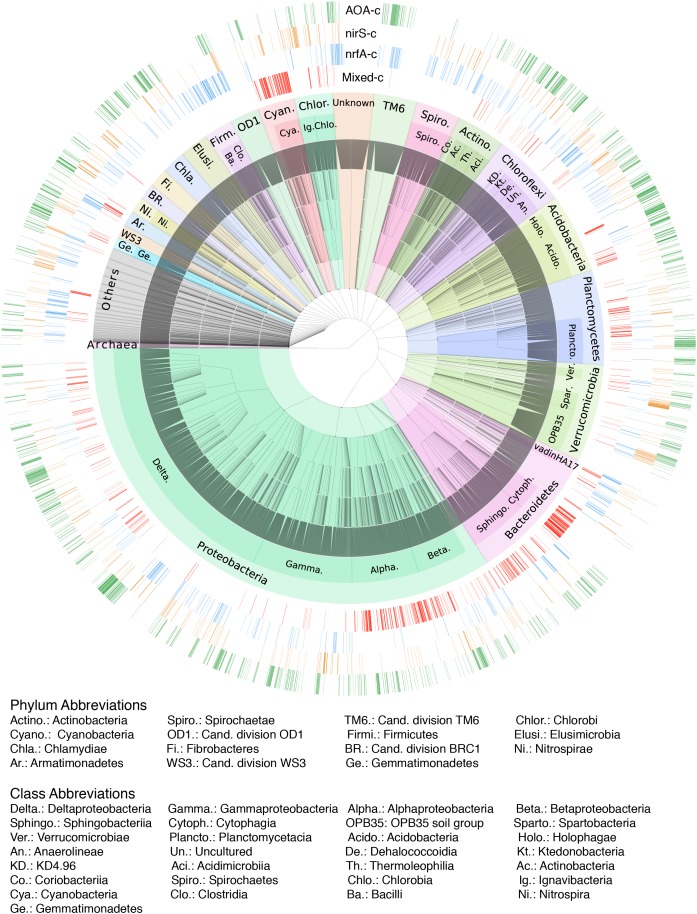
Hierarchical taxonomic classification of OTUs found in all surveyed lakes. Major bacterial phyla are indicated by shaded areas, while dominant classes within each phylum are labelled. External rings show OTUs that are exclusive indicators (adjusted-*p* < 0.001) of each of the four N-transformation functional sample clusters, as delimited by the dominant N-functional gene abundances.

The ordination of the OTU composition constrained by the N-functional gene abundances resulted in a pattern of four distinct sample clusters (Figure 5), similar to that obtained in an unconstrained ordination (Supplementary Figure S6). The four clusters consisted of samples associated with a high relative abundance of *nrfA*, *nirS*, AOA, or a combination of the rest of the targeted genes. Classification of samples into the four clusters using *k*-means followed by indicator species analysis resulted in approximately 29% of OTUs being identified as exclusively associated with samples from a single cluster (Figure 6 and Supplementary Tables S4, S5). Approximately 12 % of OTUs were significant indicators of the AOA sample cluster and found across a wide range of different bacterial taxa. By contrast, 6% of OTUs were significant indicators of the mixed N-transformation cluster, with large numbers of indicators concentrated within the phyla Cyanobacteria, Bacteroidetes, and Planctomycetes, as well as Alpha- and Betaprotoebacteria classes. Similarly, 6% of OTUs were associated with samples in the *nrfA* cluster and were classified into Firmicutes, Bacteroidetes, Actinobacteria, and Chloroflexi phyla, and Epsilon- and Deltaproteobacterial classes. Finally, 4% of OTUs were exclusive indicators of samples in the *nirS* cluster and were found across a large number of bacterial taxa, similar to the AOA sample cluster.

## Discussion

### The DNRA-Denitrification Gradient

The gradient of *nrfA* to *nirS* dominance was the main pattern of variation in the N-transforming microbial communities of the benthic habitats. From an ecosystem perspective, this gradient indicates a shift from habitats with a higher potential for internal N_r_ cycling via DNRA, and thus retention of N_r_ in the system, to those in which loss of N_r_ from the lake is more likely through denitrification. The environments with the lowest ratios of denitrifying to DNRA nitrite reductase genes were characterised by variables indicating refractory OM with high C/N, lower oxygen diffusion and lower redox potentials. This was particularly the case in the deepest part of the deep lakes (maximum depth ≥ 25 m) and in the deeper regions of the reduced elodeid sediments. In agreement with our result, previous work in a tropical high-altitude oligotrophic lake has shown *nrfA* abundance to be highest in the deepest part of the hypolimnion with anoxic conditions during late stratification (Pajares et al., 2017), and more reduced conditions favoured DNRA over denitrification in Australian estuaries (Kessler et al., 2018). Increased C/N ratio may favour DNRA over denitrification (Kraft et al., 2014).

The highest genetic potential for denitrification, based on *nirS* abundance, was detected in shallower and less oligotrophic lakes, where DIN levels were lower in the water column due to higher primary productivity. These lakes also have lower C/N ratios (Supplementary Table S1). Generally, lake autochthonous OM is fresher and of higher quality (e.g., lower C/N), and is a substantial proportion of total OM in lakes with a lower ratio of the catchment to the lake area. This fresh OM can be used as electron donors for denitrifiers, as demonstrated in several aquatic ecosystems [eutrophic lakes (Chen et al., 2012; Gardner et al., 2017), streams (Barnes et al., 2012; Stelzer et al., 2014), wetlands (Dodla et al., 2008), and oceans (Van Mooy et al., 2002)]. Oxygen levels in upper sediments of shallow and productive lakes likely fluctuate to a greater degree than those observed in habitats dominated by DNRA, thereby favouring organisms with facultative anaerobic respiration pathways such as denitrification (Wittorf et al., 2016; Chen J. et al., 2017). The *nirS* denitrifiers along the DNRA-denitrification gradient were associated with *nosZ* clade II N_2_O reduction and AOA communities involved in ammonia oxidation, whereas denitrifier communities present in lithic biofilms dominated by *nirK*-types were associated with *nosZ* clade I N_2_O reduction and AOB. These patterns indicate that different N transformation networks developed in these habitats even when in both cases exhibited potential for linked nitrification and denitrification.

The overall high proportion of denitrifying nitrite versus nitrous oxide reductase genes (∼30 *nir*:*nosZ* ratio on average) suggests a dominance of partial denitrification, especially in productive habitats dominated by *nirS* denitrifier communities (i.e., *nirS*-cluster showed *nir*:*nosZ* higher ratios compared to the other clusters, WMW test, *p* < 0.01). This observation agrees with Castellano-Hinojosa et al. (2017) who found high N_2_O/N_2_ emissions in a productive, shallow warm Mediterranean mountain lake, as well as Myrstener et al. (2016) who demonstrated that addition of nitrate, phosphorus and labile C to sediments from a boreal lake resulted in higher relative N_2_O production compared to addition of nitrate alone. Other studies have shown that higher *nir*:*nosZ1* ratios in the sediments of boreal lakes were associated with hypolimnion N_2_O excess, as well as increased phosphate and nitrate concentrations (Saarenheimo et al., 2015a). Thus, productive sites could favour partial denitrifiers that survive anoxic periods. Before arriving to the atmosphere, N_2_O might be consumed in the hypolimnion of deep lakes. *NosZ*-harbouring bacteria have been found in the hypolimnion of boreal lakes (Peura et al., 2018). However, in the sediments studied, the higher *nir*:*nosZ* ratios were found in shallower lakes. in which N_2_O may easily reach the atmosphere (Dore et al., 1998). Further studies accounting for real N_2_O emissions could corroborate our conjecture.

Nitrite-dependent anaerobic methane oxidation (N-DAMO, Simon and Klotz, 2013) is a potential alternative to DNRA, denitrification and anammox nitrite consumption. N-DAMO has been found as a key driver of methane oxidation in nitrate-rich lakes (Deutzmann et al., 2014) and reduced sandy riverbeds (Shen et al., 2019). In these habitats, bacteria related to Candidatus *Methylomirabilis oxyfera*, known to perform this pathway, were abundant. In our study, we detected only two OTUs of low relative abundance (0.02%) classified as Candidatus *Methylomirabilis*. Nonetheless, OTUs belonging to the genus *Methylocaldum* were more abundant (∼0.3%), and were significant indicators of the ‘*nirS*’ cluster. These findings are similar to those of a survey of methane oxidation in Indian reservoirs (Naqvi et al., 2018), where low relative abundance of NC10 bacteria capable of N-DAMO (0.003–0.022%) was found; whereas Type I aerobic methanotrophs, which include *Methylocaldum* and other members of the Methylococcaceae family, were predominant and co-occurred with a diverse community of potential *nirS* type denitrifiers. Other Methylococcaceae are partial denitrifying aerobic methanotrophs with N_2_O as the end product (Kits et al., 2015a,b). Overall, these results, added to the high ratio of *nir* to *nosZ* gene abundance, suggest that N_2_O emissions are the most likely endpoint of nitrite reduction in *nirS*-cluster sediments, independent of the pathway. Further studies are required to elucidate the importance and distribution of methane dependent processes in mountain lakes and to evaluate their role as a bypass of partial denitrification.

### The Idiosyncratic Lithic Biofilms

Microbial N-transforming guilds in the lithic biofilms differentiate from those in the sediments. Gene abundance results indicate a complex N-transformation structure in this habitat, consisting of processes demanding both oxic and anoxic conditions, which suggest highly structured microbial communities in relatively short spatial distances. The idiosyncratic nature of the guild composition (e.g., *nosZI*, *hdh*, *nirK*) declines as the productivity of the lake increases; *nirS* becomes more prominent compared to *nirK*, and the N-transforming communities are more similar to the upper sediments. The high abundance of *nirS* differs from the dominance of *nirK* previously found in another study of epilithic biofilms from a subset of the same lakes. A main difference between the two studies is that in Vila-Costa et al. (2014) only sampled the upper side of the cobbles, whereas we sampled both sides. Differences in ammonia oxidisers between the upper (light-side) and lower (dark-side) sides of cobbles have been previously reported (Merbt et al., 2017). The higher relative abundance of *hdh* and *nosZ* genes indicates that N loss in the form of N_2_ could be higher in the lithic biofilms compared to the other benthic habitats in the studied lakes.

### Archaeal Nitrification Hotspots

The positive δ^15^N signals observed in samples from the lower sediment layers of the isoetid rhizosphere and the rocky littoral sediments of the alpine lakes support the view of them as nitrification hotspots (Mariotti et al., 1981), likely performed by AOA as suggested by the *amoA* gene abundance. Nitrification in rocky littoral sediments could be quantitatively more relevant than the isoetid rhizosphere given that rocky littorals occupy large areas in alpine lakes. Nevertheless, the archaeal *amoA* densities observed in the lower isoetid sediment layer were nearly 100-fold higher than in the rocky sediment samples with the highest AOA abundance. Sediments close to the roots of isoetids are episodically well-oxygenated due to the release of oxygen through roots during photosynthetic periods (Sand-Jensen et al., 1982), which increases the interface between oxidised and reduced sediments where the NH_4_^+^ oxidation occurs. Intensive nitrification would result in high NO_3_^-^ accumulation in the sediment porewater (maxinun 2 mM; Catalan et al., 1994). However, this is likely transient as the NO_3_^-^ concentration in the overlying water column was negligible, suggesting a close coupling of nitrification and denitrification in this habitat (Vila-Costa et al., 2016). Indeed, there was a positive correlation (*r* = 0.68, *p* < 0.001) between nitrification and denitrification gene abundances in the isoetid sediments of Plan lake. Lakes at a higher altitude, Contraix and Gelats de Bergús, also showed significant correlations between nitrification and denitrification gene abundances for deep and littoral habitats (*r* = 0.48, *p* = 0.02 and *r* = 0.44, *p* = 0.08, respectively), suggesting that nitrification and denitrification are also linked in the nitrification hotspots.

### Linking the Taxonomic Distribution and Functional Potential

Each of the four main N-transforming communities consists of a highly diverse and distinct consortium of co-occurring bacterial taxa, based on the large number of indicator OTUs detected. However, taxonomic classification does not necessarily predict functioning, as different prokaryotic traits may be conserved at different phylogenetic depths (Martiny et al., 2015). While ammonia oxidation and anammox capacities are restricted to only a few lineages, denitrification and DNRA are widely distributed across the phylogeny (Graf et al., 2014; Welsh et al., 2014). Thus, many of the indicator OTUs identified are likely not directly involved in each N-transformation pathway. However, the high degree of similarity observed between unconstrained and functional gene-constrained OTU-based ordinations indicates that shifts in the genetic potential of different N-transformation processes are tightly linked to changes in the overall prokaryotic community structure, which itself is shaped by differences in environmental conditions across habitats.

Links between taxonomic composition and functional potential has been observed in previous works in lakes based on metagenomes or sequencing of functional genes. The occurrence of several proteobacterial families, in particular, Rhodobacteraceae (Alphaproteobacteria), Methylococcaceae (Gammaproteobacteria), and Burkholderiales, Comamonadaceae and Rhodocyclaceae (Betaproteobacteria) has been shown to be strongly associated with denitrification gene presence or abundance (Vila-Costa et al., 2014; Peura et al., 2015; Saarenheimo et al., 2015b; Castellano-Hinojosa et al., 2017; Chen R. et al., 2017). These taxa were also highly abundant in samples within the *nirS*-denitrifier and mixed functional gene communities, which included *nirK*-type denitrifiers. Moreover, metagenomic studies of boreal lakes water columns have identified *nosZ* sequences originating from Myxococcales (Deltaproteobacteria) and Sphingobacteriaceae (Bacteroidetes) in the hypolimnion near the oxycline (Peura et al., 2015, 2018). Many organisms within these families are known to possess the clade II variant of the *nosZ* gene (Hallin et al., 2018). Accordingly, a large proportion of indicator OTUs for the mixed N-cycling communities, which includes the clade II *nosZ* variant, were also classified as belonging to these families. The 4th most abundant genus in the studied lakes is *Anaeromyxobacter*, one member of this Deltaproteobacteria genus is *A. dehalogenans* a chemodenitrifier, an organism that combines chemical chemodenitrification reactions and enzymatic reaction(s) to reduce NO_3_^-^ to N_2_O or N_2_, without having denitrifying nitrite reductases codified by *nirS* or *nirK*, also performs DNRA and Fe-reduction (Onley et al., 2018). *Rhodoferax* (Beta-) and *Desulfomonile* (Delta-) possible chemodenitrifiers (Onley et al., 2018) were also common genus present. There are other eubacteria non-proteobacteria taxa also carrying denitrifying genes (Graf et al., 2014) present in our samples (e.g., Actinobacteria).

Operational taxonomic units associated with samples in the *nrfA* cluster were classified as Firmicutes, Epsilon- and Deltaproteobacteria (Campylobacterales and *Anaeromyxobacter*, *Desulfovibrio*, and *Geobacter*, respectively), Bacteroidetes (Bacteroidia), Actinobacteria (Coriobacterales and Corynebacterales) and Chloroflexi (Anaerolineaceae), all these taxa include microbes that are known to carry *nrfA* (Welsh et al., 2014).

Regarding the ammonia oxidisers, the primers used in the 16S rRNA sequencing mainly target bacteria, but also pick up Euryarchaeota (Takahashi et al., 2014). Therefore, OTUs assigned to Thaumarchaeota were not detected, although AOA hotspots could be identified based on qPCR data. Nitrosomonadaceae was the most common AOB, with 89 OTUs classified as being similar to uncultured members of this family. *Nitrospirae* was the only identified nitrite oxidising bacteria (NOB) in our samples. There was a likely coupling between AOA and NOB, as suggested by the correlation between archaeal *amoA* genes and the relative abundance of Nitrospirae members in general, and *Nitrospira* in particular, in the AOA-cluster samples (*r* = 0.65, *p* = 0.0005; *r* = 0.47, *p* = 0.02, respectively). This coupling has previously also been found in grasslands (Simonin et al., 2015), agricultural soils (Jones and Hallin, 2019), and sediments of an Andean mountain lake (Parro et al., 2019). Comammox *Nitrospira* could be important in the nitrifying hotspots found in the present study, as suggested by previous studies in other surface-attached oligotrophic habitats (Kits et al., 2017; Pjevac et al., 2017; Fowler et al., 2018). For anaerobic ammonia oxidation, OTUs belonging to the “*Candidatus* Anammoximicrobium” (Khramenkov et al., 2013) was the only taxa present with demonstrated anammox capacity. However, more bacteria within the numerous uncultured Planctomycetes detected in our samples could potentially perform anammox.

## Conclusion

**FIGURE 7:**
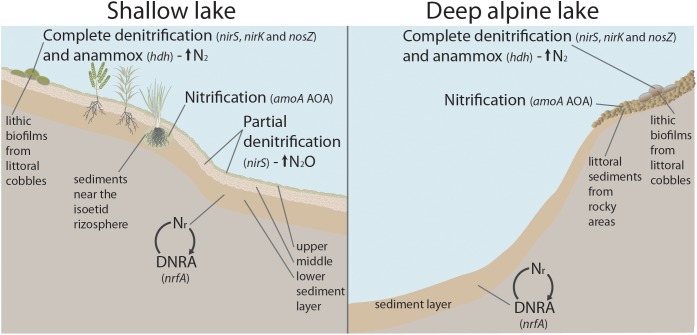
Conceptual sketch of a shallow, mountain lake with macrophytes **(left)**, and a deep alpine lake **(right)**. The benthic habitats with the genetic potential for the dominant N-cycling process(es) and fate of N (N_r_ recycling, or N_2_ and N_2_O emission) are indicated. The dominant N functional gene(s) in each habitat are shown in italics. Abbreviations: N_r_, reactive nitrogen; AOA, ammonia-oxidising archaea; anammox, anaerobic ammonium oxidation; DNRA, dissimilatory nitrate reduction to ammonium.

The N-transforming guild composition in benthic habitats of mountain lakes is complex and deeply embedded in the overall prokaryotic community. There is a high positive correlation among all the genes, and they all generally increase with OM. The dominant pathways change depending on the habitat and productivity of the lake (Figure 7). The fate of nitrite is the main diverging point differentiating the N-transforming guilds. The genetic potential for DNRA dominate in the deep part of the lakes and the lower sediment layers, which indicates recycling of the N_r_. By contrast, the denitrifying *nirS* nitrite reduction potential prevails in the upper layer of the sediments in the shallow, warmer and more productive lakes, which indicates a loss of N_r_. Emissions of N_2_O and N_2_ are likely spatially segregated within lakes, with lithic biofilms being candidates for preferential N loss as N_2_ as they show a more balanced gene abundance of nitrous oxide reductases (*nosZ*I+II) and anammox (*hdh*) in relation to NO-forming nitrite reductases (*nirS*+*nirK*). The more productive and *nirS-*dominated habitats may be a main source of N_2_O because of the striking excess of this gene over the ones of the final steps of complete denitrification unless another bypass process is relevant (e.g., N-DAMO). There may be two types of nitrifying-denitrifying coupled community types in the benthic habitats of mountain lakes. The first is based on nitrification by AOA coupled to Nitrospirae (NOB) and denitrification by *nirS*-denitrifiers, with hotspots in the rocky littoral sediments of the lakes above treeline and the sediments near the isoetid rhizosphere. The second includes AOB coupled to *nirK*-type denitrifiers reducing nitrite and *nosZ*I-N_2_O reduction in the lithic biofilms. Overall, our results point out two types of potential response to high atmospheric N deposition in these lakes. In highly oligotrophic lakes, there will be an accumulation of N_r_ because of the predominance of internal N_r_ recycling via DNRA. In less oligotrophic lakes, generally with macrophyte growth, the N_r_ deposition loads may be more effectively directed toward N gas release to the atmosphere via denitrification.

## Ethics Statement

The authors declare that the present study does not involve human or animals, that they have all the licenses, and that they follow all the rules for sampling in the Aigüestortes National Park.

## Author Contributions

CP-L and JCat contributed to the study design. LC and CP-L carried out sampling. CP-L, CJ, and JCal carried out the lab work and data analysis. JCat, SH, LC, and EC contributed to reagents, materials, and analysis tools. CP-L and JCat wrote the manuscript. All authors substantially contributed to commenting and revising it.

## Conflict of Interest Statement

The authors declare that the research was conducted in the absence of any commercial or financial relationships that could be construed as a potential conflict of interest.
