# Supplementary material for: The DNRA-Denitrification Dichotomy Differentiates Nitrogen Transformation Pathways in Mountain Lake Benthic Habitats
Source: Front Microbiol. 2019 Jun 4;10:1229. doi: 10.3389/fmicb.2019.01229 (PMC6558203; doi:10.3389/fmicb.2019.01229)
Supplement: Supplementary file 5 [file Data_Sheet_1.docx]

**SUPPLEMENTARY MATERIAL for**

**The DNRA-denitrification dichotomy differentiates nitrogen transformation pathways in mountain lake benthic habitats**

Carlos Palacin-Lizarbe^1^, Lluís Camarero^2^, Sara Hallin^3^, Christopher M Jones^3^, Joan Caliz^2^, Emilio O Casamayor^2^, and Jordi Catalan^1, 4^

^1^CREAF, Campus UAB, Cerdanyola del Vallès, Spain, ^2^Center for Advanced Studies of Blanes, (CEAB–CSIC), Girona, Spain, ^3^Swedish University of Agricultural Sciences, Department of Forest Mycology and Plant Pathology, Uppsala, Sweden, ^4^CSIC, Cerdanyola del Vallès, Spain

**Supplementary tables:** Environmental (Table S1) and gene abundance (Table S2) data by lake and habitat, OTUs exclusive indicators of one cluster (Table S4), and most influential OTUs of the prokaryotic community ordinations (com-gen-RDA and com-PCA, Table S6) are in separate excel files.

**Current file content:**

- **Table S3**. Primers and thermal cycling conditions for quantification of 16S rRNA and N-functional genes, and Illumina pyrosequencing of *16S* rRNA.
- **Table S5.** Primary indicator taxa of the microbial community associated with each N-transforming functional cluster, indicated by the dominant gene and process and the characteristics of the habitat where they were detected.
- **Figures S1-6.**
  1. Abundance of the accounted N-functional per sediment DW.
  2. Sediment profiles of *nrfA* and archaeal *amoA* abundance.
  3. Unconstrained principal component analysis ordination of the N-functional gene abundance (gen-PCA).
  4. Site scores of the gen-env-RDA and com-gen-RDA.
  5. Mean size of the sediment particles by habitat and lake and gene-ordination clusters.
  6. Principal component analysis using the Hellinger distance of the prokaryotic community (com-PCA).

**Table S3.** Primers and thermal cycling conditions for quantification of 16S rRNA and N-functional genes, and Illumina pyrosequencing of *16S* rRNA.

| **Process/Taxa** | **Genes**  Primer names | **Sequences** (5’-3’) | **References** | **Thermal cycling** | | **Efficiency** (%) | |  |
| --- | --- | --- | --- | --- | --- | --- | --- | --- |
| **Bacteria** | **16S rRNA** |  |  |  | |  | |  |
|  | **Sequencing** |  |  | (98 C, 3 min) x 1 | |  | |  |
|  | Pro341F | CCTACGGGNBGCASCAG | Takahashi et al. (2014) | (98 C, 30 s; 55 C, 30 s; 72 C, 30 s) x 25 | |  | |  |
|  | Pro805R | GACTACNVGGGTATCTAATCC |  | (72 C, 10 min) x 1 | |  | |  |
| **Bacteria** | **16S rRNA** |  |  | (95 C, 7 min) x 1 | |  | |  |
|  | 341F | CCT ACG GGA GGC AGC AG | Lopez-Gutierrez et al. (2004) | (95 C, 15 s; 60 C, 30 s; 72 C, 30 s; 80 C, 30 s) x 40 | | 90 | |  |
|  | 534R | ATT ACC GCG GCT GCT GGC A |  | (95°C, 15 s;(60 to 95° C, 10 s, increment 0.5°)), x 1 | |  | |  |
| **Denitrification** | ***nirK*** |  |  | (95 C, 7 min) x 1 | |  | |  |
| Nitrate reduction | nirK F1aCu | ATCATGGTSCTGCCGCG | Hallin and Lindgren (1999) | (95 C, 15 s; (63 C – 58 C, -1 /cycle), 30 s; 72 C, 30 s) x 6 | | 83 | |  |
|  | nirK R3Cu | GCCTCGATCAGRTTGTGGTT |  | (95 C, 15 s; 58 C, 30 s; 72 C, 30 s; 80 C, 30 s) x 35 | |  | |  |
|  |  |  |  | (95°C, 15 s;(60 to 95° C, 10 s, increment 0.5°)), x 1 | |  | |  |
| **Denitrification** | ***nirS*** |  |  | (95 C, 7 min) x 1 | |  | |  |
| Nitrate reduction | nirS cd3aFm | AACGYSAAGGARACSGG | Throbäck et al. (2004) | (95 C, 15 s; (65 C – 60 C, -1 /cycle), 30 s; 72 C, 30 s) x 6 | | 76 | |  |
|  | nirS R3cdm | GASTTCGGRTGSGTCTTSAYGAA |  | (95 C, 15 s; 60 C, 30 s; 72 C, 30 s; 80 C, 30 s) x 35 | |  | |  |
|  |  |  |  | (95°C, 15 s;(60 to 95° C, 10 s, increment 0.5°)), x 1 | |  | |  |
| **Denitrification** | ***nosZ*I** |  |  | (95 C, 7 min) x 1 | |  | |  |
| Nitrous oxide reduction | 1840F | CGC RAC GGC AAS AAG GTS MSS GT | Henry et al. (2006) | (95 C, 15 s; (65 C – 60 C, -1 /cycle), 30 s; 72 C, 30 s) x6 | | 79 | |  |
|  | 2090R | CAK RTG CAK SGC RTG GCA GAA |  | (95 C, 15 s; 60 C, 30 s; 72 C, 30 s; 80 C, 30 s) x 35 | |  | |  |
|  |  |  |  | (95°C, 15 s;(60 to 95° C, 10 s, increment 0.5°)), x 1 | |  | |  |
| **Denitrification** | ***nosZ*II** |  |  | (95 C, 7 min) x 1 | |  | |  |
| Nitrous oxide reduction | nosZII-F | CTI GGI CCI YTK CAY AC | Jones et al. (2013) | (95 C, 15 s; 54 C, 30 s; 72 C, 30 s; 80 C, 30 s) x 40 | | 65 | |  |
|  | nosZII-R | GCI GAR CAR AAI TCB GTR C |  | (95°C, 15 s;(60 to 95° C, 10 s, increment 0.5°)), x 1 | |  | |  |
| **Anammox*** | ***hdh*** |  |  | (95 C, 7 min) x 1 | |  | |  |
|  | hzocl1F1 | TGY AAG ACY TGY CAY TGG | Schmid et al. (2008) | (95 C, 15 s; 52.5 C, 30 s; 72 C, 30 s; 77 C, 30 s) x 35 | | 88 | |  |
|  | hzocl1R2 | ACT CCA GAT RTG CTG ACC |  | (95°C, 15 s;(60 to 95° C, 10 s, increment 0.5°)), x 1 | |  | |  |
| **DNRA**** | ***nrfA*** |  |  | (95 C, 5 min) x 1 | |  | |  |
|  | nrfAF2aw | CAR TGY CAY GTB GAR TA | Welsh et al. (2014) | (95 C, 15 s; 57 C, 30 s; 72 C, 30 s) x 7 | | 70 | |  |
|  | nrfAR1 | TWN GGC ATR TGR CAR TC | Mohan et al. (2004) | (95 C, 15 s; 52 C, 35 s; 72 C, 35 s; 80 C, 15 s) x 35 | |  | |  |
|  |  |  |  | (95°C, 15 s;(60 to 95° C, 10 s, increment 0.5°)), x 1 | |  | |  |
| **Nitrification** | ***amoA*** |  |  | (95 C, 5 min) x 1 | |  | |  |
| (archaeal) | crenamoA23F | ATG GTC TGG CTW AGA CG | Tourna et al. (2008) | (95 C, 15 s; 55 C, 30 s; 72 C, 40 s; 80 C, 10 s) x 35 | | 84 | |  |
|  | crenamoA616R | GCC ATC CAT CTG TAT GTC CA |  | (95°C, 15 s;(60 to 95° C, 10 s, increment 0.5°)), x 1 | |  | |  |
| **Nitrification** | ***amoA*** |  |  | (95 C, 5 min) x 1 | |  | |  |
| (bacterial) | AmoA1F | GGG GTT TCT ACT GGT GGT | Rotthauwe et al. (1997) | (95 C, 15 s; 55 C, 30 s; 72 C, 40 s; 80 C, 10 s) x 35 | | 85 | |  |
|  | AmoA2R | CCC CTC KGS AAA GCC TTC TTC |  | | (95°C, 15 s;(60 to 95° C, 10 s, increment 0.5°)), x 1 | |  | |
| *Anammox: Anaerobic ammonium oxidation. **DNRA: Dissimilatory nitrate reduction to ammonium | | | | | | | |  |

| **Table S5.** Primary indicator taxa of the microbial community associated with each N-transforming functional cluster, indicated by the dominant gene and process and the characteristics of the habitat where they were detected. |
| --- |

| **Characteristic N-functional gene(s) and process(es) of the N-transforming cluster** | **Habitat** | **Main indicator taxa of the associated microbial community** |
| --- | --- | --- |
| ***nirS***, denitrification (nitrite reduction) | All sediment habitats of the less oligotrophic, shallower and warmer lakes.  Sediments with high organic matter content and low C/N ratio. | Candidate divisions TA06 and GOUTA4  Chlorobi (Ignavibacteriales & SJA-28)  Elusimicrobia (Endomicrobia)  Fibrobacteres (TG3-1)  Spirochaetae (genus *Spirochaeta*)  Nitrospirae (Thermodesulfovibrionaceae & 4-29)  Planctomycetes (Candidatus genus *Anammoxomicrobium*)  Proteobacteria: Gamma- (genus *Methylocaldum* and species *Achromatium oxaliferum*)  Verrucomicrobia (S-BQ2-57 soil group) |
| **nrfA**, DNRA (nitrite reduction) | Deep part of all lakes, littoral of low-altitude (montane) lakes and the deepest sediment layers.  Anoxic and fine grain sediment with low δ13C and high C/N ratio. | Actinobacteria (OPB41, PeM15, and Coriobacteriaceae)  Armatimonadetes (SJA-176)  Bacteroidetes (Bacteroidia)  Candidate divisions OP3, OP8, SR1 and WCHB1-60  Chloroflexi (Dehaloccoidia & Anaerolinaceae)  Cyanobacteria, non-photosynthetic (Melainabacteria order Gastranaerophilales)  Firmicutes  Fusobacteria (uncultured Leptotrichiaceae genus)  Proteobacteria: Delta- (*Syntrophorhabdus* and Syntrophaceae) and Epsilon- (Campylobacterales)  Spirochaetae (PL-11B10) |
| **Mixed** (**nirK**, **nosZI**, **nosZII**, denitrification (nitrate reduction, nitrous oxide reduction); **hdh**, anammox (hydrazine oxidation), and **amoA AOB**, nitrification (ammonium oxidation)) | Lithic biofilms.  Highly organic thin aggregates with low δ15N and high δ13C. | Cyanobacteria: photosynthetic class Cyanobacteria (especially subsection III family I (genus *Leptolyngbya,* *Phormidium* and *Chamaesiphon*) and subsection IV)  Acidobacteria (genus *Blastocatella*)  Armatimonadetes (Armatimonadales)  Bacteroidetes (mainly Cytophagales, but also Sphingobacteriia (Chitinophagaceae, Saprospiraceae, NS11-12 marine group, and class SM1A07)  Chlorobi (Chlorobiaceae)  Deinococcus-Thermus (Deinococcaceae)  Planctomycetes (genus *Gemmata*, *Pirellula* and SM1A02)  Proteobacteria: mainly Alpha- (especially Caulobacterales, Rhizobiales, Rhodospirillales (Acetobacteraceae), Rickettsiales and Sphingomonadales); but also Beta- (Burkholderiales) and Gamma- (Pseudomonadales) |
| **amoA** (**AOA**), nitrification (ammonium oxidation) | Deep sediments near the rhizosphere of isoetid macrophytes.  Rocky littoral sediments of lakes in the alpine belt.  Sediments with high δ15N. | Acidobacteria (subgroups 1, 2, 5, 6, 7, 10, 12, 13, 15 and 17)  Actinobacteria (orders Acidimicrobiales, Gaiellales and class MB-A2-108)  Bacteroidetes (Sphingobacteriales (families AKYH767, CWT CU03-E12, PHOs-HE51 and S15-21))  Candidate divisions TM6 and WD272  Chlamydiae (Chlamydiales, mainly families Parachlamydiaceae, Simkaniaceae and cvE6)  Chloroflexi (classes Ktedonobacteria, JG30-KF-CM66, JG37-AG-4, P2-11E, S085 and TK10)  Cyanobacteria (non-photosynthetic, Melainabacteria (order Obscuribacterales))  Elusimicrobia (lineages (IIa, IIb, IIc, IV), FCPU453 and MVP-88)  Fibrobacteres (order 258ds10)  Gemmatimonadetes (Gemmatimonadaceae)  Nitrospirae (family 0319-6A21 and uncultured members of the genus *Nitrospira*)  Planctomycetes (OM190, Pla4 lineage, and an uncultured Genus of Planctomycetaceae)  Proteobacteria: Alpha- (Rhodospirillales (DA111, I-10 and KCM-B-15)); Beta- (uncultured Nitrosomonadaceae genus), Gamma- (genus Beggiatoa and orders Legionellales (especially genus *Aquicella*, *Coxiella* and *Legionella*), Xanthomonadales, and NKB5); and Delta- (GR-WP33-30 and myxococcales (especially genus *Haliangium*, and families Cystobacteraceae, Polyangiaceae, Sandaracinaceae and mle1-27)  Verrucomicrobia (order Chthoniobacterales mainly from family DA101 soil group) |

**Figure S1**. Sum of the accounted N-functional gene copies standardised to 16S rRNA gene copy abundance. Lakes are arranged from left to right by increasing water-column DIN level (see Table 2 for lake abbreviations and features) Hereafter, boxplots depict the interquartile range (box), median value (line), 1.5 x interquartile range (whiskers), and outliers (points).

**Figure S2**. A) *NrfA* abundance sediment profiles. B) Archaeal (AOA) *amoA* abundance sediment profiles. Note that *amoA* abundance (X-axis) is square root transformed. Dashed lines connect sediment samples from the same core but different layer. All samples are from Plan Lake except two from Redon de Vilamòs Lake (star symbol).


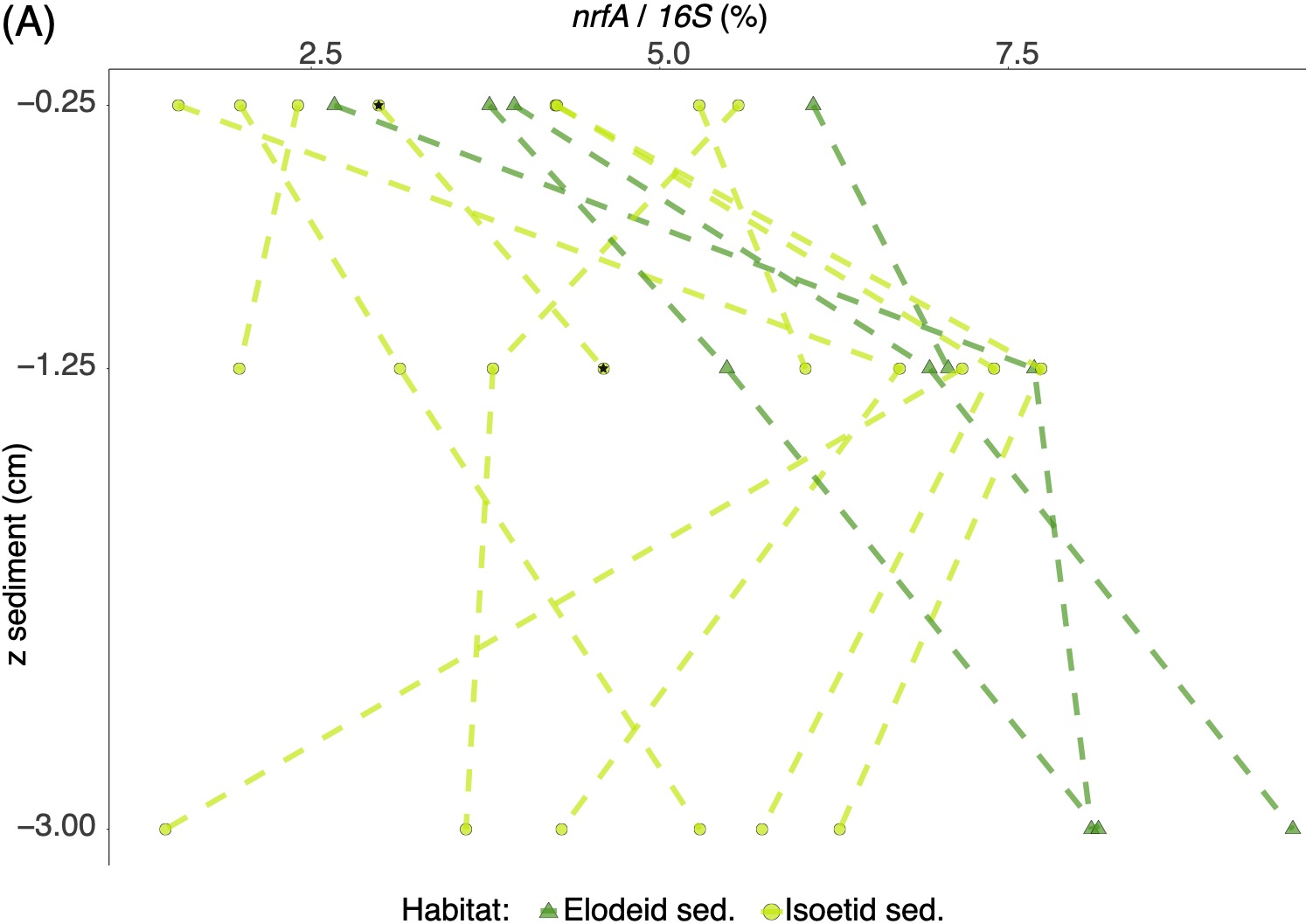


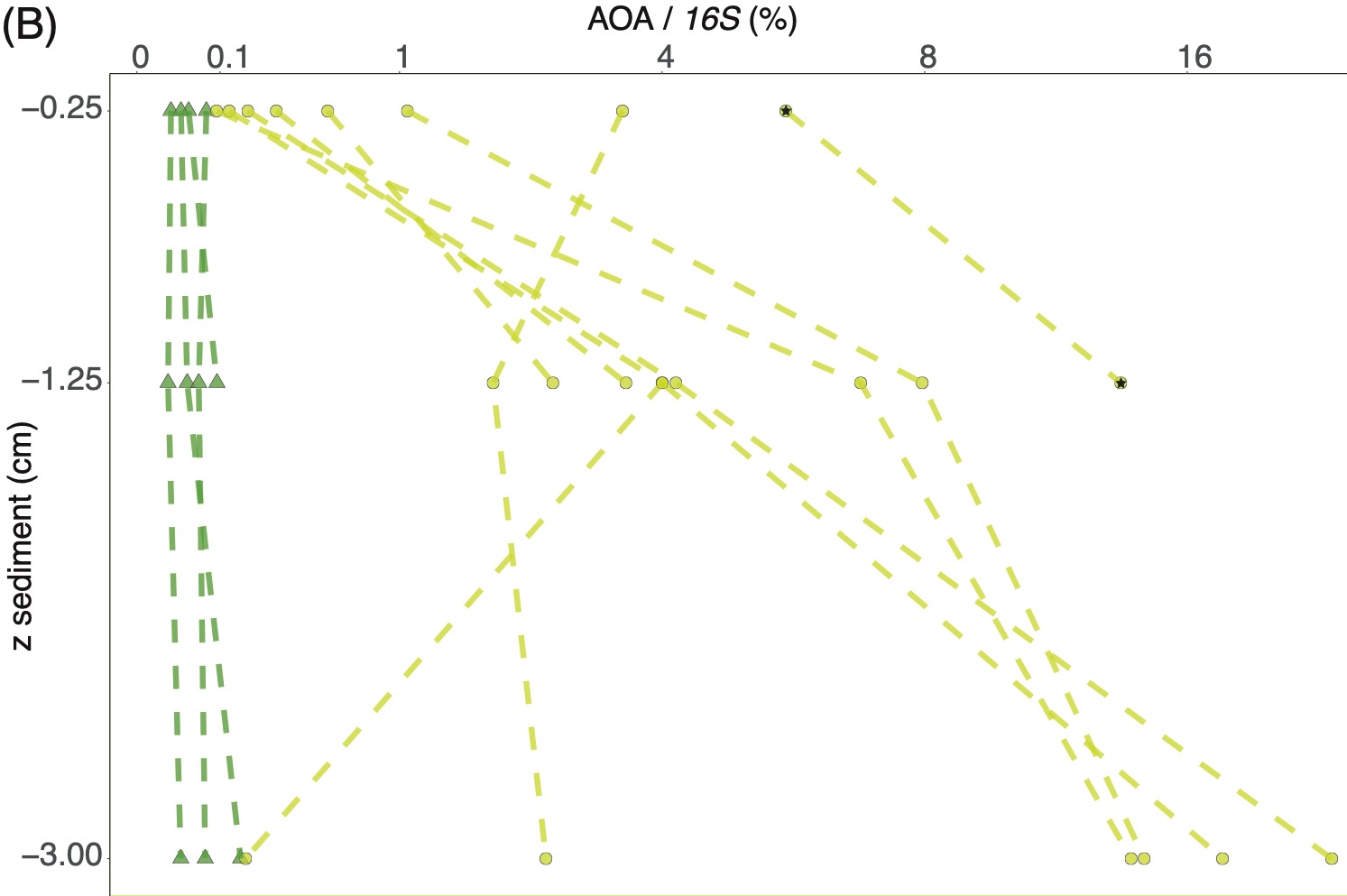


**Figure S3**. Unconstrained ordination of the N-functional gene abundances resulted from a principal component analysis using Hellinger distance (gen-PCA). A) Biplot of the 1^st^ and 2^nd^ and (B)) 1^st^ and 3^rd^ main gradients. The PCA axes’ length is scaled to the variation explained.

**
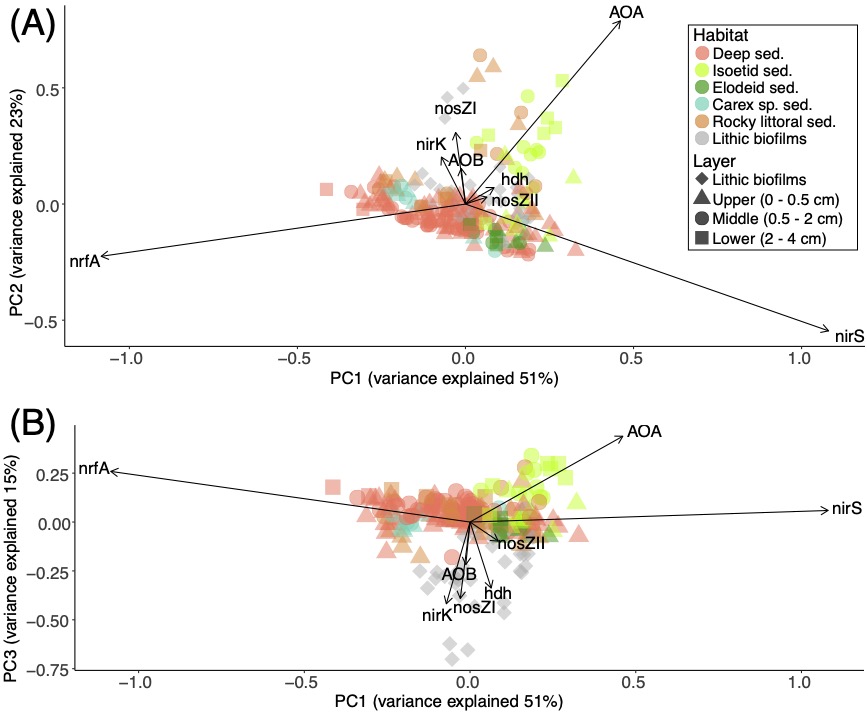
**

**Figure S4**. Site scores of gen-env-RDA (A, B) and com-gen-RDA (C, D). Symbol labels correspond to the lake abbreviations (see Table 2).

**
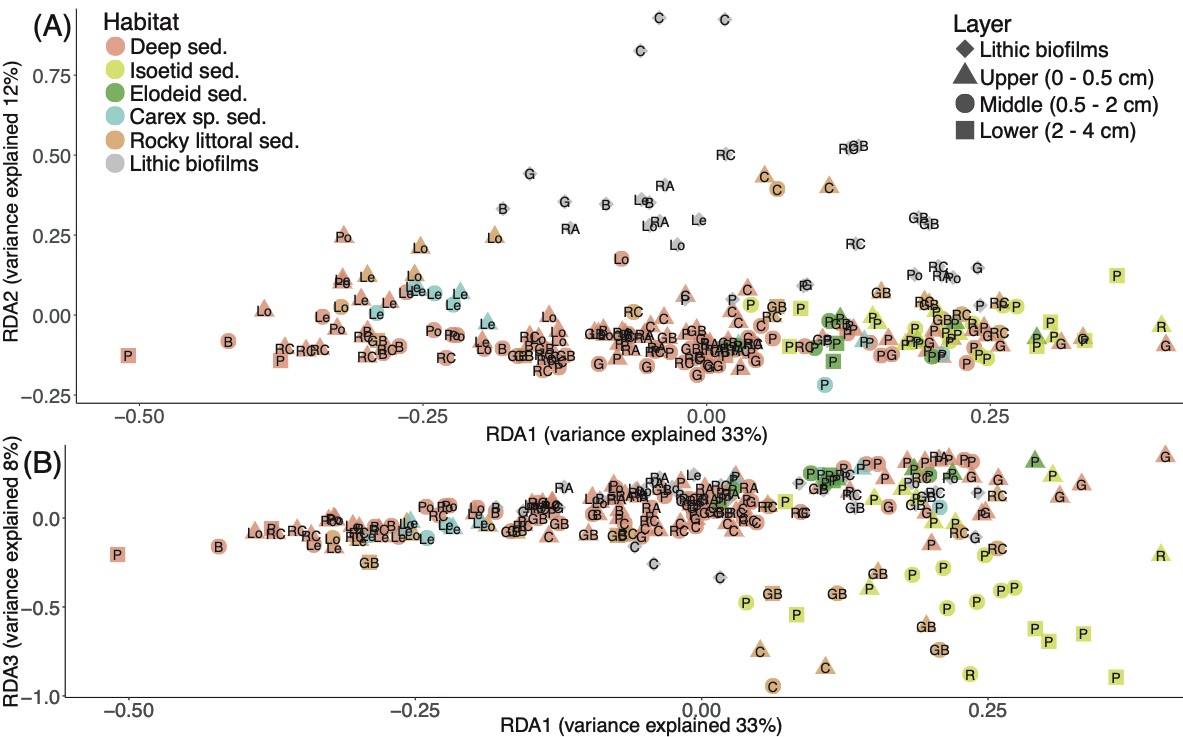
**

**
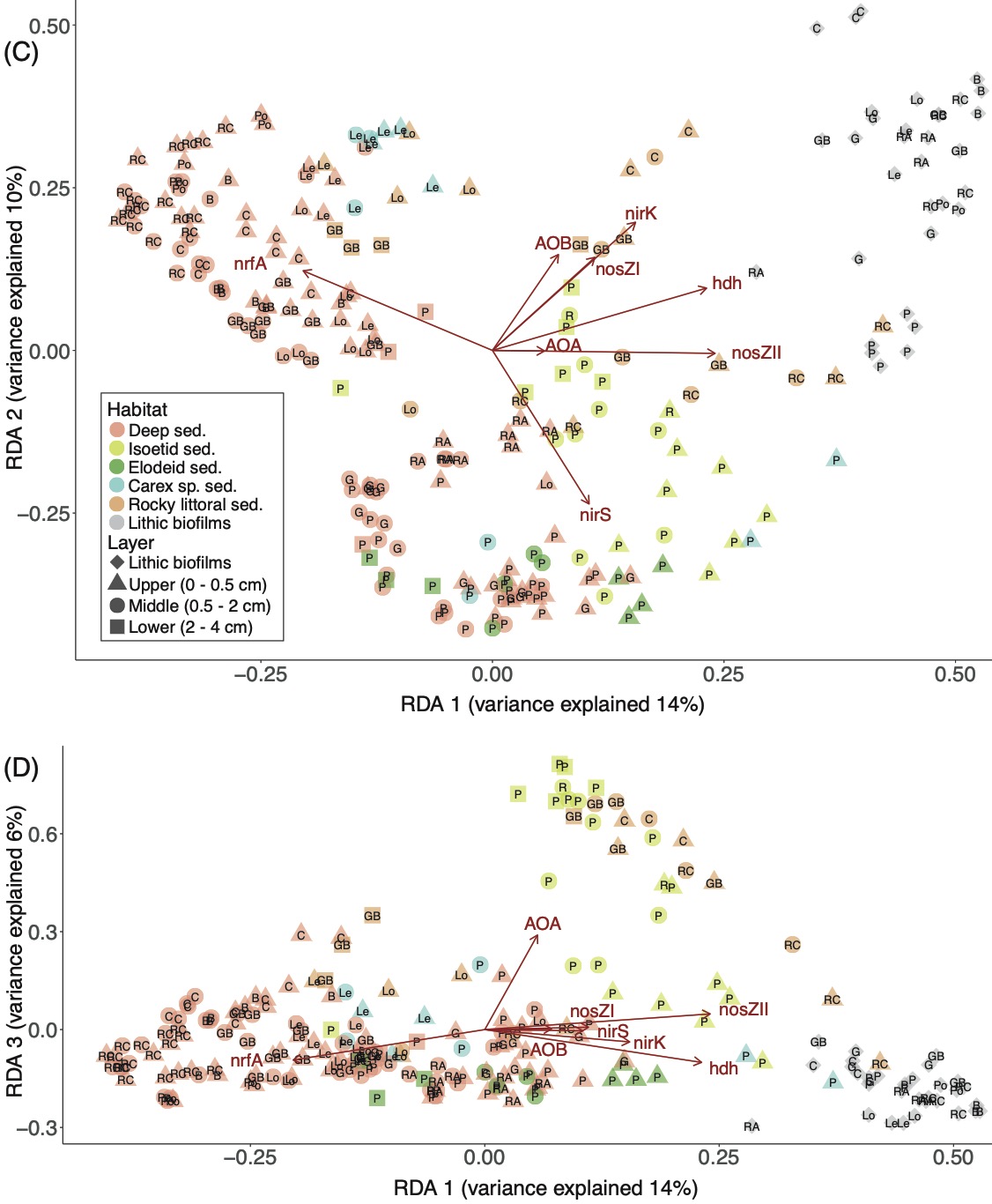
**

**Figure S5.** Mean size of sediment particles by habitat and lake (A) or gene-rich cluster (B). Note: Granulometry of lithic biofilms was not analysed. Size of sediment particles differed between the gene-rich clusters (Kruskal-Wallis test χ^2^ = 52, p <0.001, followed by a Wilcoxon-Mann Whitney test p < 0.05, differences are indicated in the cluster labels by “(letter)”).

|  |
| --- |

**Figure S6**. Unconstrained ordination of the prokaryotic community resulted from a principal component analysis based on the OTUs abundance using Hellinger distance (com-PCA). Biplot of the 1^st^ and 2^nd^ main gradients. Note that PCA axes sizes are proportional to the explained variation. Only the most influential OTUs for each gradient are shown (black arrows and ID, Table S6)

**
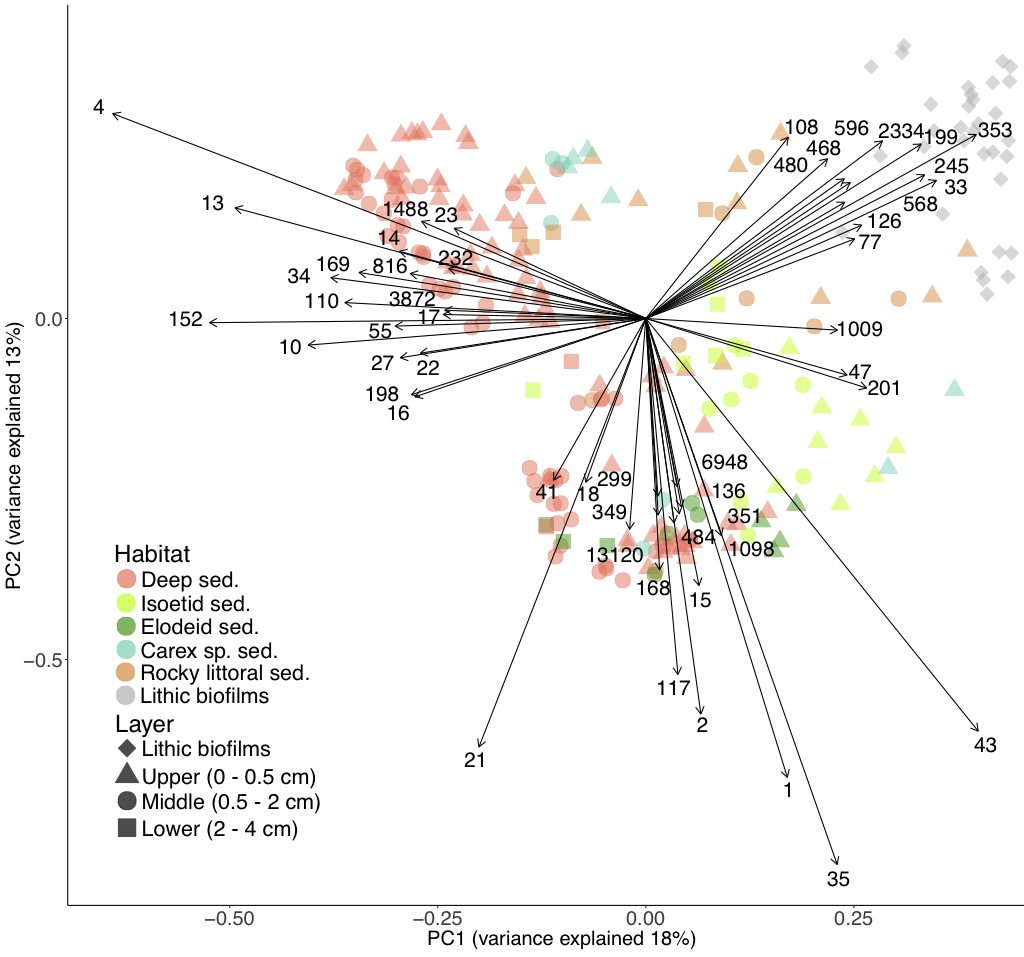
**
